# Botanical Characters of the Asarum Canadense

**Published:** 1819-10

**Authors:** 


FOR THE LONDON MEDICAL AND PHYSICAL JOURNAL.

### *Botanical Characters of the Asarum Canadense.*

IN the case of tetanus successfully treated by Dr. Firth, related in the last number of your Journal, a decoction of *asarum canadense* was given to the patient, amongst the other remedies. I believe the botanical characters of that plant have not been given at any time in your Journal; if it be so, you will perhaps be pleased with the following for insertion.

*London; Sept. 8th.* PHILOPHYTON.

---

ASARUM, cl. *dodecandria*, ord. *monogynia*, LIN. Cl. v. *dicotyledons*, ord. *aristoloches*, JUSSIEU. Cl. *apétales*, sect. i. TOURNEFORT.

*Gen. char.*—Calyx three or four cleft, superior; corolla none; anthers growing to the middle of the filaments. Capsule coriaceous, crowned.

*Spec. char.*—Leaves two, reniform; calyx woolly, cleft to the base; its segments spreading at top. Grows in old woods and mountainous tracts. Flowers from May to July. Michaux styles it "vix distinctum ab Europæo," with regard to form; but its taste is somewhat different, being intermediate between that of ginger and the aristolochia serpentaria. In its qualities it appears to resemble the latter.

